# Temporal interaction of suicidal ideations and behaviors with loneliness in persistent depressive disorder – a feasibility study using ecological momentary assessment

**DOI:** 10.1007/s00406-024-01931-8

**Published:** 2024-11-02

**Authors:** Johannes Wolf, Stephan Goerigk, Franziska Midderhoff, Gerrit Burkhardt, Markus Bühner, Stephan Köhler, Peter Falkai, Andrea Jobst, Frank Padberg, Matthias A. Reinhard

**Affiliations:** 1 Department of Psychiatry and Psychotherapy, LMU University Hospital, Nussbaumstr. 7, 80336 Munich, Germany; 2 DZPG (German Center for Mental Health), Partner Site, Munich, Germany; 3 Charlotte Fresenius Hochschule, Munich, Germany; 4 Department of Psychology, Ludwig-Maximilians-University Munich, Munich, Germany; 5 Department of Psychiatry and Neurosciences, Charité –Universitätsmedizin Berlin, Campus Mitte, Berlin, Germany

**Keywords:** Suicidality, Loneliness, Ecological momentary assessment, Depression

## Abstract

Individuals with persistent depressive disorder (PDD) are at increased risk for suicidality. Suicidality may be precipitated by loneliness. However, their temporal interplay in PDD has not been studied. We conducted a feasibility study using ecological momentary assessment (EMA) to measure short-term courses of suicidality and loneliness in 20 inpatients with PDD and current suicidality. EMA adherence of 13 completers was 81.3%. Suicidal ideations and loneliness varied with one standard deviation over three to six hours. This pilot study confirmed the feasibility of EMA in PDD and provided new insights in dynamics of suicidality and loneliness informing future study designs.

**Supplementary Information:**

The online version contains supplementary material available at 10.1007/s00406-024-01931-8.

## Highlights

We confirmed the feasibility of EMA in PDD.

Suicidal ideation and loneliness varied with one SD in three to six hours.

Strong contemporaneous associations between SIB and loneliness were observed.

**Supplementary Information:**

The online version contains supplementary material available at 10.1007/s00406-024-01931-8.

## Introduction

Suicide is a major source of mortality in severe mental disorders [1, 2]. Among such conditions, chronic depression, i.e. persistent depressive disorder (PDD) according to DSM-5, is associated with greater disability compared to episodic depression [3] and shows high levels of suicidal ideation and behaviors (SIB) [4, 5].

SIB are associated with loneliness in PDD [4]. An association between SIB and loneliness has also been shown in a meta-analysis based mostly on community samples [6]. A theoretical background for this association is provided by Joiner’s interpersonal theory, according to which thwarted belongingness, i.e. loneliness, and burdensomeness, i.e. the feeling of being a burden for others, increase the risk for SIB [7]. However, data on the short-term development of SIB and risk factors is lacking [2]. A deeper understanding of the short-term interplay between SIB and loneliness could theoretically inform interventions for suicide prevention.

Ecological momentary assessment (EMA) provides a tool to study short-term symptom dynamics. EMA has been widely applied in ambulatory assessment [8, 9], but fewer EMA studies have been conducted in clinical samples, none in PDD [9–11].

To establish EMA as a novel method for measuring symptom dynamics in PDD, this pilot study tested the feasibility of EMA in PDD patients with current SIB and investigated the variability and covariance of SIB and loneliness over short periods of time in a pilot dataset. We aimed to gain first data informing the following research questions: (1) Over which time periods do SIB and loneliness vary? (2) Do SIB and loneliness covary over time? (3) Do loneliness patterns at one time point predict SIB at the following time point?

## Methods

### Participants

Participants were inpatients with PDD reporting current suicidal ideation (SI) who were treated at the LMU Hospital Munich, Department for Psychiatry and Psychotherapy (see supplement for exclusion criteria). All participants provided written informed consent prior to inclusion. Diagnoses were assigned according to DSM-5 criteria via structured clinical interviews (SCID-5-CV/ -PD). The study was approved by the ethical board of the medical faculty of LMU Munich (Ref. 22–0299).

### Procedure

EMA was applied four times daily for one week at the beginning and one week at the end of a five-week study period to maximize measurement time without overburdening participants. Participants underwent extensive clinician-rated and self-report assessments at baseline which are detailed in the supplement.

### EMA

EMA measures were applied at randomized time points from 8 a.m. to 8 p.m. local time via the movisensXS application (movisens GmbH, Karlsruhe, Germany) which runs on Android systems only. At every prompt, patients rated (1) passive SI, active SI, SI intensity, and suicidal behaviors (SB); (2) momentary loneliness including feelings of loneliness, perceived social and emotional isolation; (3) burdensomeness; and (4) momentary affect. Items were taken from existing EMA item repositories, adapted from previous studies in the field [12], and modified based on validated questionnaires. Items were scored on visual analogue scales with scores ranging from “0” to “100”. Item formulations can be found in the supplement.

### Statistics

Analyses were performed using SPSS Version 26 and R version 4.3.2 (R Core Team 2023). To address research question (1) regarding variability over time, we used intraclass correlations (ICCs) and root mean square of successive differences (RMSSD) [13]. ICCs indicate the percentage of variability stemming from differences within vs. between individuals *on a range from zero to one*,* where one means 100% of variability in the respective variable stems from differences between individuals.* Consequently, 1-ICC represents the proportion of variability stemming from variations within individuals. The RMSSD statistic indicates variability over time between two consecutive time points. Higher values indicate more variability between two consecutive time points. RMSSD were interpreted in comparison to the standard deviation (SD) of the respective variable. *For example*,* RMSSD = SD means that the respective variable varies with one SD between two consecutive time points.* Research question (2) (covariance) was assessed using contemporaneous multilevel vector autoregressive models (mlVAR) and research question (3) was analyzed with time-lagged (lag = t-1) mlVAR Lag was set to one time point, i.e. associations between said variable at t and t-1. We did not control for multiple testing in the mlVAR given it is not feasible due to the high number of tests (for further reasoning see [14]). Statistics are described in more detail in the supplement.

## Results

### Clinical characteristics of the study sample and feasibility

Forty-one patients with PDD were screened, out of which 20 patients (48.7%) agreed to participate. There were no significant differences in age or sex between patients who participated vs. who did not. The main reason for not participating in 66.6% was an incompatible mobile operating system. 33% did not participate, reporting being too burdened or not feeling like participating. At baseline, participants were 38.8 ± 6.1 years old and 50.0% were female. Depression was severe on average. 7 participants (35%) showed current active SI, and 13 (65%) showed current passive SI. One patient showed current SB. Clinical and sociodemographic characteristics at baseline are reported in Table 1.

**Table 1:** Clinical characteristics at baseline

Variable	Study sample (*n* = 20)	Completers (*n* = 13)	Non-completers (*n* = 7)	*p*-value
Demographics				
Gender, female, n (%)	10 (50.0)	8 (40.0)	2 (28.5)	0.175
Age, mean (SD)	38.8 (6.1)	40.6 (14.0)	35.5 (17.6)	0.429
Marital status, in partnership, n (%)	6 (30)	5 (38)	1 (14)	0.210
Depression				
BDI-II, mean (SD)	32.7 (10.9)	28.6 (9.6)	40.2 (9.7)	**0.020**
MADRS, mean (SD)	34.9 (6.1)	32.0 (4.3)	40.1 (5.7)	**0.002**
UCLA Loneliness, mean (SD)	3.2 (0.8)	2.9 (0.8)	3.8 (0.6)	**0.028**
UCLA feelings of loneliness, mean (SD)	3.2 (0.8)	2.9 (0.9)	3.7 (0.5)	0.075
UCLA emotional isolation, mean (SD)	3.0 (1.0)	2.6 (0.8)	3.6 (1.1)	**0.043**
UCLA social isolation, mean (SD)	3.4 (0.7)	3.1 (0.7)	4.0 (0.5)	**0.012**
C-SSRS worst lifetime suicidality				
No lifetime SI, n (%)	0 (0)	0 (0)	0 (0)	0.536
Lifetime passive SI, n (%)	0 (0)	0 (0)	0 (0)	
Lifetime unspecific active SI, n (%)	9 (45.0)	5 (38.5)	4 (57.1)	
Lifetime SI, plan & intent, n (%)	11 (55.0)	8 (61.5)	3 (42.9)	
No lifetime SB, n (%)	4 (20)	3 (23.1)	1 (14.3)	0.115
Lifetime SB without SA, n (%)	8 (40)	7 (53.8)	1 (14.3)	
Lifetime SA, n (%)	8 (40)	3 (23.1)	5 (71.4)	
C-SSRS worst current suicidality				
No Current SI, n (%)	0 (0)	0 (0)	0 (0)	0.699
Current passive SI, n (%)	13 (65)	9 (69.2)	4 (57.1)	
Current unspecific active SI, n (%)	7 (35)	4 (30.8)	3 (42.9)	
Current SI, pan & intent, n (%)	0 (0)	0 (0)	0 (0)	
No current SB, n (%)	19 (95)	13 (100.0)	6 (85.7)	0.643
Current SB, n (%)	1 (5)	0 (0)	1 (14.3)	

*Note*. BDI-II = Beck Depression Inventory 2nd Version; MADRS = Montgomery-Asberg Depression Rating Scale; UCLA = UCLA Loneliness Scale; C-SSRS = Columbia Suicide Severity Rating Scale; SI = suicidal ideation; SB = suicidal behavior; independent t-Test was used for metric variables; Mann-Whitney-U Test was used for ordinal variables; Chi–squared test was used for nominal variables

Out of 20 participants who started the study, 5 (25%) dropped out after the first week because of technical problems with the EMA application, which did not reinitiate surveys in the second week of assessment. One participant withdrew, reporting that the study was too burdensome, and a second participant was transferred to another hospital for medical reasons. Non-completers reported higher scores of depressive symptoms (self-rating: *p* = .020, clinician-rating: *p* = .002) and loneliness (*p* = .028). With respect to adherence, the remaining 13 participants completed 81.3% of automated prompts.

### Variability over time of suicidality and loneliness

**Fig. 1:**
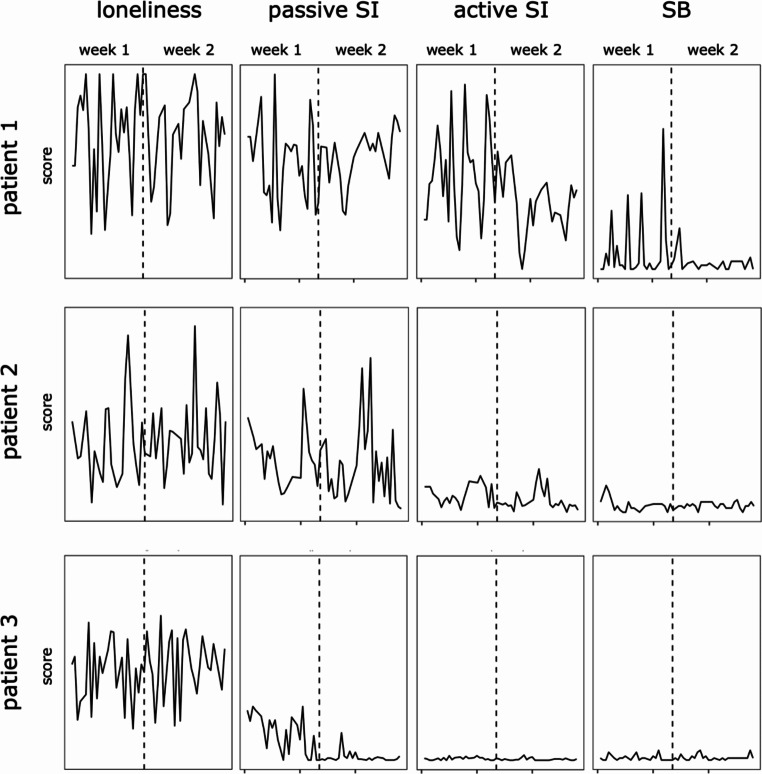
Individual EMA scores of loneliness and SIB *Note*. Higher scores indicate more severe symptom burden; Loneliness = feelings of loneliness; SI = suicidal ideations; SB = suicidal behaviors; here we show three single cases to illustrate the inter-individual variation of our findings: Patient 1 was a 19-year-old female student who reported symptom onset at the age of 5, had two suicide attempts and underwent five inpatient treatment periods in childhood and adolescence. At baseline, she showed moderate to severe depression (Montgomery-Asberg Depression Rating Scale, MADRS score: 27; Beck Depression Inventory 2nd Version, BDI score: 34), high levels of loneliness (UCLA Loneliness Scale, UCLA sum score: 66), and active SI including thoughts about specific methods but neither intent nor SB according to the Columbia Suicide Severity Rating Scale (C-SSRS). EMA scores of loneliness and SIB of this patient were high with fluctuations over both measurement weeks including peaks of SB in week 1. Patient 2 was a 63-year-old female disability pensioner who had symptoms since the age of 16, without lifetime suicide attempts and reported two previous inpatient treatment periods. At baseline, depression was moderate to severe (MADRS score: 30; BDI score: 35); loneliness was high (UCLA sum score: 72); and passive SI were reported in the C-SSRS. EMA scores showed a moderate momentary symptom burden with visually corresponding peaks in loneliness and passive SI but low variability in active SI, and no relevant variability in SB. Patient 3 was 30-year-old male shop assistant reporting symptoms since the age of 14, no previous suicide attempts, and two periods of inpatient treatment. At baseline, he reported moderate depression (MADRS score: 25; BDI score: 27), moderately high loneliness (UCLA sum score: 57), and passive SI. EMA scores of this patient showed moderate momentary loneliness with some passive SI in the first week but neither active SI or SB nor peaks in symptom burden over the measurement period

RMSSD statistics indicated variability in both composite scores and individual items. Affect items ‘arousal’ and ‘valence’, as well as loneliness items ‘feelings of loneliness’ and ‘perceived social isolation’ varied for approximately one SD between consecutive time points. Scores for passive SI, active SI, and intensity of SI varied with about 0.5 SD between consecutive time points. SB showed little variance between consecutive time points. Analyzing ICCs, approximately 70–80% of the variation in affect items and loneliness scores (except ‘perceived emotional isolation’), 40% of variance in SI scores, and 10% of variance in SB scores can be attributed to differences within individuals rather than between individuals. Descriptive statistics for EMA as well as ICC and RMSSD estimates can be found in Table 2. To illustrate differences of symptom progression, EMA scores of 3 selected patients are depicted in Fig. 1.

### Temporal interplay between loneliness and suicidality

**Fig. 2:**
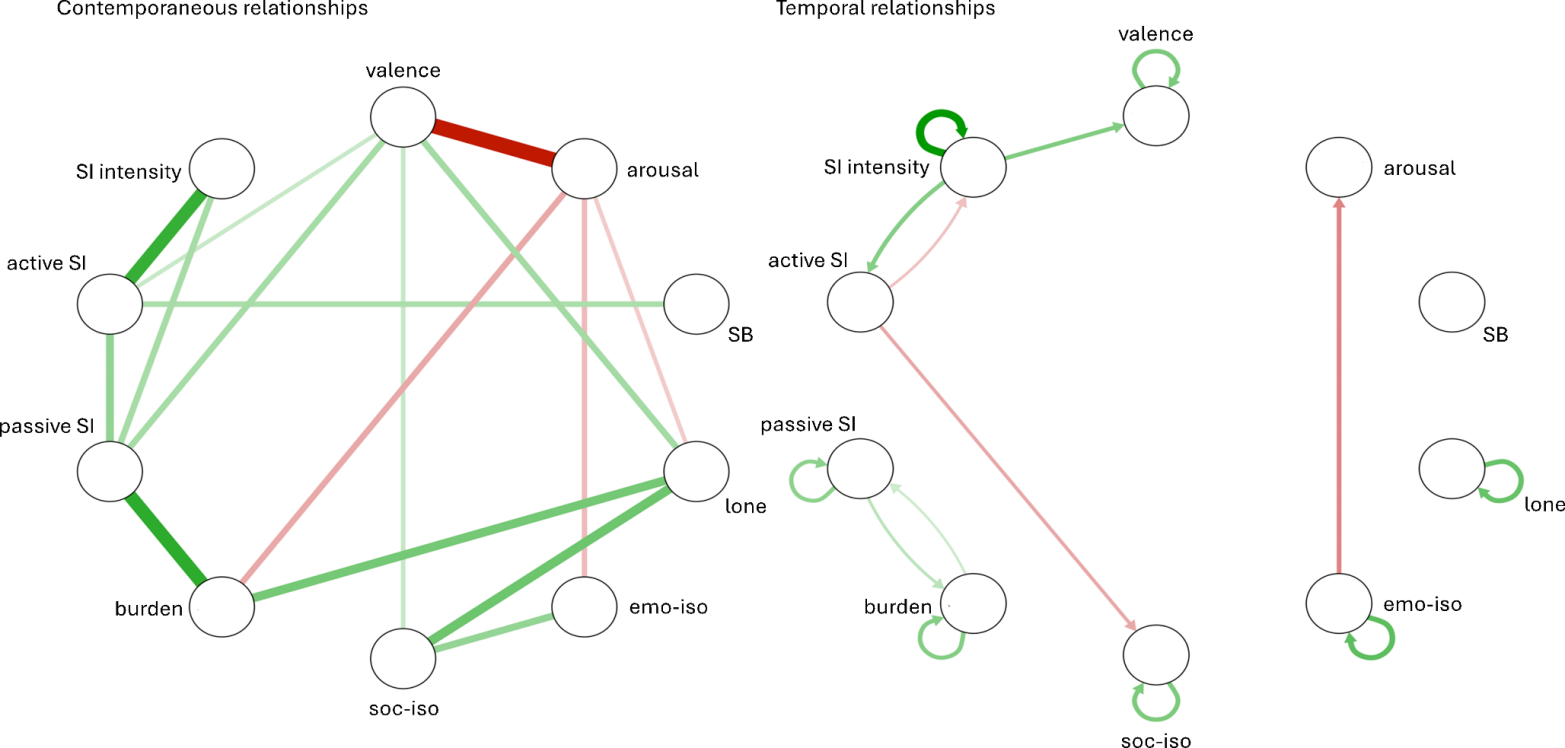
Symptom associations over time, results of multilevel vector autoregressive models *Note*. Circles represent constructs; lines between constructs represent significant associations; arrows indicate the direction of the underlying association; green lines represent positive associations, red lines represent negative associations; the thicker the line, the stronger the association; SI = suicidal ideation; SB = suicidal behavior; emo-iso = emotional isolation; soc-iso = social isolation; lone = loneliness feelings; burden = burdensomeness

Using mlVAR, we observed significant contemporaneous associations between several variables. Significant positive association paths were found between aspects of loneliness, passive SI, active SI, and the intensity of SI. SB was weakly positively associated with active SI and the intensity of SI. Temporal relationships were observed from the intensity of SI to valence and active SI. Negative temporal relations were observed from emotional isolation to arousal and from SB to valence. Temporal autoregressive effects were observed in all variables except active SI. Figure 2 shows symptom networks (contemporaneous and temporal) as calculated using mlVAR.

## Discussion

This study provides first evidence for the feasibility of EMA assessing temporal dynamics of SIB and loneliness in a high-risk PDD sample. Affect and loneliness ratings varied significantly within a few hours, i.e., on average three hours, while passive as well as active SI and their intensity seem to vary within longer periods, i.e. on average six hours. Variance in SB was low. Our pilot data reveal strong contemporaneous associations between affect, loneliness, and SIB, while temporal relations were mostly driven by autoregressive effects.

Few studies have used EMA for assessing SIB in clinical populations [10–12, 15] and to our knowledge, none of these studies have assessed SB. Additionally, EMA has not yet been used in PDD. Due to technical constraints of our EMA application (i.e., exclusive compatibility with Android operating systems), a considerable number of patients could not participate. In addition, we observed a relevant proportion of non-completers because of technical issues. Higher levels of depressive symptoms and loneliness might have been another factor for non-completion. There is little published evidence regarding participation and adherence to EMA studies in the field [8]. Our findings seem in line with other research reporting technical issues [16] and lower adherence in more severely depressed individuals [17]. Completers answered 81.3% of automated prompts, comparable to overall compliance in the field [8]. In contrast to other studies in inpatient settings [12, 15, 18], participants were not financially incentivized in the present study, speaking to the scalability of the presented approach.

While the present findings clearly support the need for assessing SIB and loneliness in short periods, i.e., hours rather than days, our findings regarding variability over time stand partly in contrast with findings from other studies reporting significant variability of SI over periods of one to few hours in patients with MDD [12, 15, 18]. This contrast may be explained by differences in size and characteristics of the respective clinical samples, e.g. the chronicity of depressive symptoms in PDD as well as a mostly stable clinical environment in the present sample. Inter-individual differences of the three individual cases showcased in Fig. 1 may demand an in-depth investigation of intra-individual effects in future studies.

**Table 2:** Descriptive and variability statistics of EMA

	Descriptive statistics				Variability statistics			
Variable	M	SD	Range	Skew	ICC	RMSSD	RMSSD range	
Suicidality								
Passive SI	38.03	27.91	0-100	0.21	0.63	16.13	4.27–37.14	
Active SI	13.83	17.46	0–95	1.51	0.62	9.31	0.85–20.86	
SI intensity	19.78	19.00	0-100	1.33	0.57	9.70	1.84–19.26	
SB	7.32	18.78	0–82	3.05	0.93	3.34	0.37–17.09	
Loneliness								
Feelings	43.92	23.84	0-100	0.23	0.26	22.14	11.16–33.9	
Emotional isolation	52.88	27.68	0-100	-0.32	0.69	15.95	9.14–24.26	
Social isolation	50.89	23.26	0-100	-0.12	0.19	22.72	13.2–33.8	
Total loneliness	49.20	19.04	0 -100	-0.05	0.34	16.58	8.71–26.46	
Affect								
Valence	52.75	21.22	0-100	0.06	0.19	20.07	11.07–31.39	
Arousal	45.36	24.75	0-100	0.23	0.24	24.02	12.58–37.21	
Burdensomeness	42.93	28.69	0-100	0.18	0.65	17.44	2.38–35.33	

*Note* M = Mean, SD = Standard Deviation, ICC = intraclass correlations, RMSSD = root mean square of successive differences, SI = suicidal ideation, SB = suicidal behavior

In line with two previous studies, we observed contemporaneous associations of EMA items related to loneliness and SI [12, 15]. Having used sampling frequencies of 4 to 10 daily assessments the data suggest that loneliness and SIB co-occur simultaneously or peak within minutes apart from each other.

Limitations of the present study include the small sample size which limits generalizability and demands cautious interpretation of significant results; a short duration of EMA which may not have captured the full symptom range; and a fixed time resolution not capturing information on symptoms occurring between 8 p.m. and 8 a.m.

In conclusion, this study provides first evidence for the feasibility of EMA in PDD. Patterns of SIB and loneliness varied significantly within three to six hours, which may inform sampling schemes of future studies. Future studies may replicate and extend the present findings in larger samples. Those studies may further optimize both the single items of EMA as well as the temporal resolution of prompts based on findings from our pilot data. Further steps will then include developing just-in-time (digital) interventions, i.e. via smartphone, that can complement current interventions to improve early recognition and prevention of SIB using EMA data.

## Electronic supplementary material

Below is the link to the electronic supplementary material.

Supplementary Material 1

## Data Availability

The data that support the findings of this ongoing study are not openly available due to reasons of sensitivity and are available from the corresponding author upon reasonable request.
