# Supplementary material for: Temporal interaction of suicidal ideations and behaviors with loneliness in persistent depressive disorder – a feasibility study using ecological momentary assessment
Source: Eur Arch Psychiatry Clin Neurosci. 2024 Nov 2;275(4):1253–9. doi: 10.1007/s00406-024-01931-8 (PMC12148998; doi:10.1007/s00406-024-01931-8)
Supplement: Supplementary file 1 — Supplementary Material 1 [file 406_2024_1931_MOESM1_ESM.docx]

### Supplement

1. **Inclusion/ Exclusion criteria**

Patients who were younger than 18 or older than 70 years of age, did not meet DSM-5 criteria for PDD, could not provide written informed consent, or showed signs of a severe somatic condition were not included.

1. **Clinical assessments**

SIB were assessed using the German version of the Columbia-Suicide Rating Scale [(Posner et al. 2011)](https://paperpile.com/c/LV8cnc/nINU). We computed (1) lifetime sum scores for SI and suicidal behavior (SB) and (2) current sum scores for SI and SB, indicating the most severe SI or SB experiences patients had during their lifetime or in the past week respectively. Loneliness was measured with the German Version of the UCLA Loneliness Scale (UCLA; Döring and Bortz 1993) including the subscales feelings of loneliness, perceived emotional isolation, and perceived social isolation. Depressive symptoms were measured with the German versions of the Beck Depression Inventory 2nd version (BDI-II, self-rating; Beck et al. 1996) and the Montgomery–Åsberg Depression Rating Scale (MADRS, observer rating; Montgomery and Asberg 1979).

1. **EMA items:**

(1) Suicidal ideations and behaviors were measured as follows: passive SI (composite score with items “*At the moment life is not worth living.*” and “*There are more reasons to die than to live at the moment*.”), active SI (composite score with items “*Currently, I am thinking about killing myself.*”, “*I am currently making plans on how to kill myself.*” and “*How strong is your desire to kill yourself?*”), intensity of SI (composite score with items *“How often do you experience suicidal thoughts at the moment? Anchors: Never to Always”* and *“I cannot stop thinking about suicide.”*), and SB (single item “*I have just made preparations or started to do something to end my life (e.g. collecting pills, writing a suicide note).*“); The safety procedure in case of SB included 1) gaining informed consent in a doctor’s visit prior to study participation including thorough information about the study proceedings and recommendations in case of acute suicidal ideation or behavior; 2) A notification in the app, that if SB was reported, patients should immediately contact their treatment team, and 3) regular visits by the treatment and study team, including nurses, psychiatrists, and psychologists to monitor suicidality.

(2) Loneliness was measured according to UCLA subscales feelings of loneliness (“*At the moment I feel lonely.*”; “*At the moment I feel left out.*”), perceived emotional isolation (composite score with items “*At the moment, there is someone I can turn to.*” and “*At the moment I feel like there is someone who really understands me.*”), perceived social isolation (composite score with items “*At the moment I have enough companionship.*” and “*At the moment I feel comfortable with my company*.”);

(3) Burdensomeness was measured with a composite score with items *“At the moment I feel useless.”* and *“At the moment I feel like a burden to others.”*;

(4) Momentary affect was measured using bipolar items to measure valence (“*My mood is… Anchors: Very bad to very good.*”) and arousal (“*I feel… Anchors: passive to active.*”).

1. **Detailed description of statistical methods**

Descriptive statistics were used to characterize the patient sample. Independent t-Tests, chi-squared tests and Mann-Whitney-U tests were used to test for differences between completers and non-completers. Intraclass correlations (ICCs) were derived from an unconditional random effects model with measurements nested within patients using the *performance* package and root mean square of successive differences (RMSSD) [(Ebner-Priemer et al. 2009)](https://paperpile.com/c/LV8cnc/tSdM). Data were detrended by regressing patient-individual time-series on time. Level stationarity was assessed for the repeated measurements of each outcome of all individual patients using the Kwiatkowski-Phillips-Schmidt-Shin (KPSS) test.
